# Feeding preterm infants with breast milk – the role of maternal pressure

**DOI:** 10.1093/eurpub/ckac131.446

**Published:** 2022-10-25

**Authors:** I Schwab, I Okumu, I Ohnhäuser, II Dresbach, I Scholten

**Affiliations:** 1 Institute of Medical Sociology, Health Services Research, and Rehabilitation Science, University of Cologne, Cologne, Germany; 2 Department of Neonatology, University Hospital Bonn, Bonn, Germany

## Abstract

**Background:**

Motheŕs own milk (MOM) is the best nutrition for preterm infants because of its preventive effects. Nevertheless, current research shows that mothers have problems getting into continuous lactation, especially after preterm birth. Breastfeeding-related pressure after prematurity has not been measured yet. It's relevance for the lactation is thus unclear, as well as the role of the NICU staff's attitudes. The aim of this study is to gain more knowledge about breastfeeding-related pressure in order to enable mothers to have a positive breastfeeding experience and to sensitise NICU staff about this topic.

**Methods:**

The written survey included mothers of preterm infants with a birth weight under 1500g and an age from 6 to 24 months at the time of the survey. Descriptive and bivariate testing was used for analyses.

**Results:**

Data of 506 mothers was included (32% response rate). One third totally agreed to perceive pressure regarding breastfeeding their child with MOM (36%). A milk volume over 500ml/day 14 days post-partum was reported in 60%. That the nutrition with MOM was promoted by the physicians in the NICU was totally agreed by 44% of the mothers. To the promotion by nurses, 50% totally agreed. Pearson Chi^2^-Test showed a significant correlation between milk volume and breastfeeding-related pressure (p = 0.005). Spearman’s correlation test showed a significant correlation between a high promotion of MOM by physicians (Spearman’s rho: -0.1150, p = 0.0109) and nurses (Spearman’s rho: -0.0949, p = 0.0362) and lower breastfeeding-related pressure.

**Conclusions:**

Our findings indicate that breastfeeding-related pressure seems to affect most of the mothers of preterm infants and correlates with lactation, even if no direction of effect can be stated. A more breastfeeding promoting NICU staff is related to lower breastfeeding-related pressure. Therefore, NICU staff should be sensitised to breastfeeding-related pressure with regard to communication with mothers.

**Key messages:**

• Noticing breastfeeding-related pressure as an important factor for mothers within their lactation process may have the potential to enhance mothers to achieve their breastfeeding goals.

• NICU staff should be aware of breastfeeding-related pressure to enable more mothers to have a positive breastfeeding experience.

